# Draft Genome Sequence of Enterococcus faecalis UMB7780, Isolated from the Female Urinary Tract

**DOI:** 10.1128/MRA.00400-20

**Published:** 2020-05-28

**Authors:** Martin Kalski, Taylor Miller-Ensminger, Adelina Voukadinova, Alan J. Wolfe, Catherine Putonti

**Affiliations:** a Department of Biology, Loyola University Chicago, Chicago, Illinois, USA; b Bioinformatics Program, Loyola University Chicago, Chicago, Illinois, USA; c Department of Microbiology and Immunology, Stritch School of Medicine, Loyola University Chicago, Maywood, Illinois, USA; d Department of Computer Science, Loyola University Chicago, Chicago, Illinois, USA; University of Maryland School of Medicine

## Abstract

Here, we present the draft genome sequence of Enterococcus faecalis UMB7780, isolated from the female urinary tract. The genome size is 3,005,901 bp, with a GC content of 37.36%, genome coverage of 179×, and an *N*_50_ score of 169,627 bp. Genome analysis identified evidence of antibiotic resistance, as well as intact prophages.

## ANNOUNCEMENT

Enterococcus faecalis is part of the normal flora of the human microbiota, including the gastrointestinal tract, oral cavity, and urinary tract (see review in reference 1). E. faecalis has been found in the urinary tract microbiota (urobiome) of individuals with and thsoe without a lower urinary tract syndrome (2–6). Enterococci are common nosocomial pathogens in humans (3–5), and *E. faecalis* and *E. faecium* cause the majority of hospital-acquired enterococcal infections (6). Likewise, *E. faecalis* is a common cause of urinary tract infections (UTI) (5, 7). E. faecalis challenges medical institutes due to its ability to rapidly acquire resistance to a majority of antibiotics (6–9). Here, we present the E. faecalis UMB7780 genome, collected from a catheterized urine sample obtained from a female patient with overactive bladder (OAB).

E. faecalis UMB7780 was isolated using the expanded quantitative urinary culture (EQUC) protocol (10) from a prior institutional review board (IRB)-approved study (University of California, San Diego, IRB no. 170077AW). The genus and species for the isolate were determined by matrix-assisted laser desorption ionization–time of flight (MALDI-TOF) mass spectrometry, following a protocol previously described (10, 11), and the sample was stored at −80°C. From this freezer stock, the isolate was streaked onto a Columbia nalidixic acid (CNA) agar plate using aseptic techniques and incubated for 24 h at 35°C with 5% CO_2_. A single colony was collected from the plate and cultured in liquid brain heart infusion (BHI) medium under the same conditions as described earlier. Genomic DNA was extracted using the Qiagen DNeasy blood and tissue kit. The kit’s Gram-positive extraction protocol was modified as follows: 230 μl of lysis buffer (180 μl of 20 mM Tris-Cl, 2 mM sodium EDTA, and 1.2% Triton X-100 and 50 μl of lysozyme) was used in step 2, and the incubation time in step 5 was reduced to 10 min. The extracted DNA was quantified using the Qubit fluorometer. The sample was sent to the Microbial Genome Sequencing Center (MiGS) at the University of Pittsburgh for sequencing. An Illumina tagmentation enzyme was used to fragment the DNA, and indices were attached using PCR. The library was then sequenced using the Illumina NextSeq 550 platform, producing 2,197,539 pairs of 150-bp reads. The raw reads were trimmed using Sickle v1.33 (https://github.com/najoshi/sickle) and assembled using SPAdes v3.13.0 with the “only-assembler” option for k values of 55, 77, 99, and 127 (12). Genome coverage was calculated using BBMap v38.76 (https://sourceforge.net/projects/bbmap/). The genome was initially annotated using PATRIC v3.6.3 (13) but then reannotated with the Prokaryotic Genome Annotation Pipeline (PGAP) v4.11 (14). PGAP annotation was used for the publicly available assembly. Unless otherwise mentioned, default parameters were used for all software tools.

The E. faecalis UMB7780 draft genome is 3,005,901 bp and assembled into 61 contigs with a genome coverage of 179× and an *N*_50_ score of 169,627 bp. The GC content is 37.36%. The PGAP annotation identified 2,802 protein-coding genes, 51 tRNAs, and 3 rRNA operons. The PATRIC annotation identified 47 genes associated with antimicrobial resistance. The ResFinder v 3.2 server predicts that UMB7780 is resistant to macrolides and tetracycline (15). The website PHASTER (16) identified 2 incomplete and 2 intact prophages within the strain’s genome. One CRISPR array was detected by the CRISPRCasFinder website (17). Additional testing and analysis of this strain will provide a better understanding of antibiotic resistance within the human urobiome.

### Data availability.

This whole-genome shotgun project has been deposited in GenBank under the accession no. JAAUVY000000000. The version described in this paper is the first version, JAAUVY010000000. The raw sequencing reads have been deposited in the SRA under the accession no. SRR11441029.
